# Efficiency of Alternative Reinforcement Methods for Wooden Ceilings and Their Ecological Aspects

**DOI:** 10.3390/ma18092032

**Published:** 2025-04-29

**Authors:** Karl Deix, Christian Huber, Josip Gogic

**Affiliations:** 1 Department of Building Material, Vienna University of Technology, 1040 Vienna, Austria; 2 Camillo Sitte Bautechnikum, 1030 Vienna, Austria; c.huber@bautechnikum.at; 3 Vienna University of Technology, 1040 Vienna, Austria; josip.gogic@wernersobek.com

**Keywords:** wooden ceilings, reinforcement, carbon-fiber-reinforced polymer, aramid-fiber-reinforced polymer

## Abstract

In the case of load increases and the refurbishment of existing buildings, it is often necessary to carry out strengthening measures on existing timber beams. When timber concrete composite (TCC) ceilings cannot be used, it is possible to reinforce the undersides of the beams with structural steel or fiber composites (aramid or carbon-fiber-reinforced polymer). This work investigates how significant effects on the load-bearing and deformation behavior can be achieved with these materials in terms of construction practice. The article is intended to show structural engineers which reinforcement measures lead to which forces, deformations, etc., and how these are utilized. This should form the basis for the planning of reinforcement measures, as it is not clear from the beginning whether AFRP, CFRP, or steel is the most suitable material. For this purpose, a comparative parameter study was carried out under practical conditions and with a variable degree of reinforcement using the corresponding formulas. The internal forces in the timber and reinforcement cross-sections, the deflection behavior, and the failure loads at the strength and design levels were calculated. It was demonstrated that, particularly for steel and carbon-fiber-reinforced polymer (CFRP) reinforcements, significant increases in the ultimate load can be achieved and the often-important deformation behavior can be significantly improved. Especially the steel variant leads to high improvements in deflection and breaking load behavior, with the base material (wood) also being utilized more economically as a result. A comparative ecological study in the form of the global warming potential showed that reinforcement methods are also advantageous from the point of view of sustainability compared to renovations with timber concrete composite slabs or new concrete slabs.

## 1. Introduction

Especially in the case of residential buildings in urban areas, it is necessary to preserve the existing substance to a large extent with regard to ecological and urban developmental aspects. Changes in use and adaptations lead to increased demands on load-bearing structures. The timber concrete composite (TCC) construction method has been established for reinforcing wooden ceilings, especially in the last three decades. The application of a thin concrete slab on existing wooden beams requires appropriate accessibility of the floor surface. If this is not possible, for example in the case of lack of construction height or the room above is not accessible for renovation, it is an option to reinforce the underside of the slab. Since this is subjected to bending tension, high-strength tension members are required for this purpose. Therefore, only materials such as reinforcement steel and fiber composites, including carbon-fiber-reinforced polymer (CFRP) laminates and aramid (AFRP) elements, can be considered.

A method often used to reinforce timber beams in existing buildings is the design of TCC ceilings, as described in [1,2]. In this case, a concrete slab is produced on the upper side, which increases the load-bearing capacity of the ceiling structure in connection with the wooden beams. The strength of the composite is essential for the interaction of the two materials. Several calculation methods can be used, such as the differential equation method according to Natter and Hoeft [3], and the gamma method. These calculation methods are compared in [4] for a practical design example.

Regarding the reinforcement of wood with steel, some literature is available. Experimental and numerical investigations are described in [5]. The dimensioning is reported in detail in [6] and numerous finite element calculations are described in [7]. In summary, the above-mentioned literature shows that steel reinforcements increase the service load by 32 to 53% and the ultimate load by 43 to 70% compared to unreinforced construction.

Reinforcement using carbon fiber (CFRP) has been studied experimentally and numerically in [8,9,10,11,12,13,14]. The influence of CFRP sheets on the load-bearing capacity of glued laminated timber beams under bending is presented in [15]. The mentioned studies show that increases in stiffness of 36 to 64 % are possible with CFRP, depending on the number of layers used. The test results were also confirmed with the FEM. State-of-the-art reviews are included in [16,17], and a study on the profitability of combining wood and CFRP is presented in [18]. Tests and FE-analysis described in [19] show that simplified models assuming a perfect connection between adherents are inappropriate for a PUR glue. Regarding the use of aramid for reinforcing wood cross-sections, there are a few studies available, as in [20,21,22]. These investigations show for various applications that reinforcement measures are also possible with aramid fibers. Basic literature on fiber reinforcement of structures can also be found in [23], especially regarding wood and CRPF in [24,25,26,27].

For this work, we referred to the studies of Blaß [28] and Luggin [29], who have dealt with reinforcements using CFRP laminates. In their investigations, the effectiveness of bonded CFRP laminates was confirmed on glulam beams in four-point bending tests. The force distribution was not investigated analytically using the equations of elastic composite theory, but rather, the effectiveness of the tension laminate was expressed as a sum via a fictitious increase in the modulus of elasticity of the plain wood beam. Furthermore, the associated failure loads were determined or calculated. Other important aspects, such as the impact and post-impact response and long-term performance and fire safety aspects of FRP Composites are covered in [30,31].

The authors of this article conducted their own tests to investigate the load-bearing behavior of wooden beams with bonded aramid and reinforcing steel bars, which are published in [32]. The basis here was formed by corresponding pull-out tests in a small format to determine the displacement modulus or joint stiffness and large bending tests to determine the deflection behavior and ultimate loads. The associated calculation models of the composite theory were also verified and confirmed with regard to their validity and applicability.

The large-scale bending tests described in [28,29,32] and their verification with different calculation methods for concrete design cases were carried out with a constant reinforcement ratio and load arrangement (four-point bending test) and the same support span in each case. In addition to these investigations, a comparative parameter study was performed in this paper with conditions close to those in practice. For a variable reinforcement ratio, it was determined to what extent significant effects on the load-bearing behavior can be achieved with reinforcement measures in the form of steel and fiber composites.

This work investigates how significant effects on load-bearing and deformation behavior can be achieved with different reinforcement materials in terms of construction practice. For this purpose, a comparative parameter study was carried out under practical conditions and with a variable degree of reinforcement using the corresponding formulas. The internal forces in the timber and reinforcement cross-sections, the deflection behavior, and the failure loads at the strength and design levels were calculated. The aim of the calculations is to show how the various reinforcements affect these parameters and which ones are decisive for a design. The results provide a decision-making basis for the selection of rehabilitation methods.

For reasons of sustainability, it is essential to consider the effects on the environment in addition to the mechanical properties when considering structural renovation measures. In Section 3.4, the global warming potential (GWP) was used to evaluate the different reinforcement methods as well as conventional renovation using TCC ceilings or concrete-only ceilings.

## 2. Materials and Methods

### 2.1. Procedure

For the design and calculation of reinforcements, the relevant characteristic values such as normal force, bending moment, utilization factor, deflection, maximum load in the service condition and at failure, and span width were determined. In the first step, the material properties were determined and a typical static system common in practice was chosen. In the second step, the corresponding exact differential equations for the elastic composite theory and engineering model for the ultimate load were found. Then, for the calculation of the characteristic values, the degree of reinforcement was varied and displayed graphically.

In composite systems, the load-bearing behavior is mainly determined by the interaction of the parameters and, thus, of the following:

- The geometry of the parts to be joined;
- Their modulus of elasticity;
- Joint stiffness;
- The strength of the composite partners;
- The static framework conditions such as span, load position, and support.

In the case of reinforcement measures for truss slabs in existing buildings, due to the large number of specified framework conditions, only the influence of the geometry and stiffness of the composite element to be applied, as well as the compliance ratios in the composite joint, can be considered.

### 2.2. Materials

With regard to the modulus of elasticity and the strengths of aramid composites, CFRP, and steel, sufficient valid data are now available for the respective standards and product specifications. Table 1 provides an overview of the materials considered in this work. These are mean values and not characteristic values.

### 2.3. Experimental Studies

**Figure 1:**
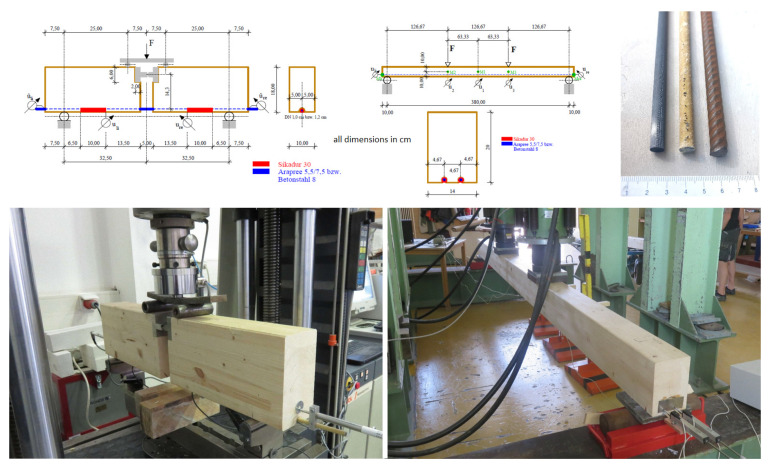
Test arrangement according to [32].

The authors of this article conducted their own tests to investigate the load-bearing behavior of wooden beams with bonded aramid and reinforcing steel bars, which are published in [32]. Special pull-out tests were carried out to determine the displacement modulus and the contact joint stiffness of small test specimens with AFRP rods (3 test specimens each with diameters: 5.5 and 7.5 mm) and reinforcing steel bars (3 test specimens with diameter: 8 mm) as the reinforcements. Figure 1, left shows an example of the test arrangement of these experiments and a scheme, which had the following parameters: Wood beam: 14/20 cm, span: 65 cm, inner span of the load points: 15 cm, lever arm between joint and reinforcement: 14.3 cm, compound length of the reinforcement: 10 cm.

Bending tests (Figure 1, right): Wood beam 14/20, span: 3.80 m, loads in the third points, two reinforcements on the bottom: 3 test specimens each AFRP: d = 5.5 mm and 7.5 mm, 3 test specimens with steel: d = 8 mm, 3 test specimens without reinforcement. The load was applied according to EN 26891 until breakage at a speed of 50 N/s. In each case, reinforcing bars with Sikadur^®^ 30 as the adhesive [37] were applied, the translational-horizontal load-displacement behavior in the glued area was measured and the joint stiffness k was derived from this. The joint stiffness was 248 N/mm^2^ for the 5.5 mm AFRP rods, 307 N/mm^2^ for the 7.5 mm AFRP rods, and 596 N/mm^2^ for the 8 mm reinforcing steel bars, with the values referring to one reinforcing element in each case.

The use of the measurement results presented here in the context of the computational simulation led to very good agreements with the measured displacement quantities from the large-scale bending tests carried out in [32]. With regard to a practical design for construction, it can be determined on the basis of the results in [32] that the simplified assumption of a rigid composite (joint stiffness k → ∞) is reasonable. This results in significant simplifications for the handling of the equal-load case when using the gamma method according to Eurocode 5 [38], derived, among others, by Heimeshoff in [39] with γ = 1.

### 2.4. Basic Equations of the Elastic Composite Theory

**Figure 2:**
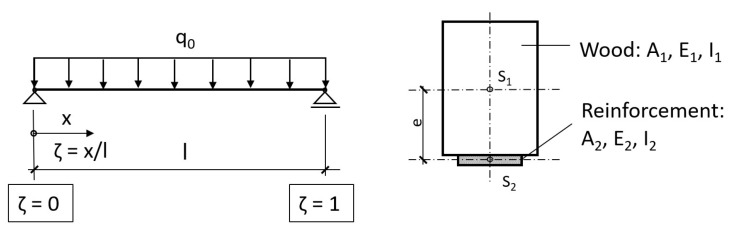
Single-span beam with uniform line load according to [3].

In elastic composite theory, it is assumed that in the composite beam partial internal forces act in the individual components (wood and laminate). Assuming linear-elastic conditions for the composite components, a differential equation system for the static quantities can be derived for the internally static indeterminate problem. The solution of these differential equations according to Natterer and Hoeft [3] leads to the determination equations for the force and displacement quantities on the system given below (Equations (1)–(9)). The formula apparatus of the strict elastic composite theory according to Natterer and Hoeft [3] for the partial internal forces and deflections used for the calculation is given as follows (Figure 2):

(1) $$w_{\left(\zeta \right)}=\frac{q_{0}\cdot l^{4}}{B}\cdot \left\{\frac{a^{2}}{1-a^{2}}\cdot \left[\frac{1}{2\cdot \lambda^{2}}\cdot \left(\zeta -\zeta^{2}\right)+\frac{1}{\lambda^{4}}\cdot \left(\frac{\cosh\left(\lambda \cdot \left[\zeta -0.5\right]\right)}{\cosh\left(\frac{\lambda }{2}\right)}-1\right)\right]+\frac{1}{24}\left(\zeta -2\cdot \zeta^{3}+\zeta^{4}\right)\right\}$$

(2) $$N_{\left(\zeta \right)}=q_{0}\cdot l^{2}\cdot \frac{a^{2}}{e}\cdot \left[\frac{1}{2}\cdot \left(\zeta -\zeta^{2}\right)-\frac{1}{\lambda^{2}}\cdot \left(1-\frac{\cosh\left[\lambda \cdot \left(\zeta -0.5\right)\right]}{\cosh\left(\frac{\lambda }{2}\right)}\right)\right]$$

(3) $$M_{i\left(\zeta \right)}=q_{0}\cdot l^{2}\cdot a_{i}\cdot \left\{\frac{1}{2}\cdot \left(\zeta -\zeta^{2}\right)\cdot \left(1-a^{2}\right)+a^{2}\cdot \frac{1}{\lambda^{2}}\cdot \left(1-\frac{\cosh\left[\lambda \cdot \left(\zeta -0.5\right)\right]}{\cosh\left(\frac{\lambda }{2}\right)}\right)\right\}$$

were

(4) $$\zeta =\frac{x}{l}$$

(5) $$\lambda =\sqrt{\left(k\cdot \frac{\left(E_{1}\cdot A_{1}+E_{2}\cdot A_{2}\right)}{E_{1}\cdot A_{1}\cdot E_{2}\cdot A_{2}}+\frac{k\cdot e^{2}}{E_{1}\cdot I_{1}+E_{2}\cdot I_{2}}\right)\cdot l^{2}}$$

(6) $$a^{2}=\frac{1}{\frac{\left(E_{1}\cdot A_{1}+E_{2}\cdot A_{2}\right)\cdot \left(E_{1}\cdot I_{1}+E_{2}\cdot I_{2}\right)}{E_{1}\cdot A_{1}\cdot E_{2}\cdot A_{2}\cdot e^{2}}+1}$$

(7) $$\frac{a^{2}}{1-a^{2}}=\frac{E_{1}\cdot A_{1}\cdot E_{2}\cdot A_{2}\cdot e^{2}}{\left(E_{1}\cdot A_{1}+E_{2}\cdot A_{2}\right)\cdot \left(E_{1}\cdot I_{1}+E_{2}\cdot I_{2}\right)}$$

(8) $$B=E_{1}\cdot I_{1}+E_{2}\cdot I_{2}+\frac{E_{1}\cdot A_{1}\cdot E_{2}\cdot A_{2}\cdot e^{2}}{\left(E_{1}\cdot A_{1}+E_{2}\cdot A_{2}\right)}$$

(9) $$a_{i}=\frac{E_{i}\cdot I_{i}}{E_{1}\cdot I_{1}+E_{2}\cdot I_{2}}$$

*w*_(*ζ*)_: deflection at the position ζ;

*N*_(*ζ*)_: normal force at the position ζ;

*M*_(*ζ*)_: moment at the position ζ;

*λ*: auxiliary value;

*i* = 1: wood;

*i* = 2: reinforcement;

*e*: distance between the individual centers of gravity;

*k*: joint stiffness;

*l*: span;

*E*_1_: modulus of elasticity for wood;

*E*_2_: modulus of elasticity for reinforcement;

*A*_1_: cross-section for wood;

*A*_2_: cross-section for reinforcement;

*I*_1_: moment of inertia for wood;

*I*_2_: moment of inertia for reinforcement;

*B*, *a*: auxiliary values.

### 2.5. Engineering Model for Calculation of Breaking Loads

The engineering model of Schatz [40] with a partially plasticized compression zone was used to determine the failure loads. The corresponding formula apparatus for timber beams with FRP laminates on the bending tensile side is shown by the following:

(10) $$\mu =\frac{A_{2}}{A_{1}}$$

(11) $$p=\frac{f_{c}}{f_{m}}+1$$

(12) $$q=\frac{f_{c}}{f_{m}}-(n-1)\cdot \mu$$

(13) $$r=2\cdot \frac{z_{L}}{h}\cdot (n-1)\cdot \mu$$

(14) $$\phi_{II}=\frac{q}{r}\cdot \left[\sqrt{{\left(\frac{p}{q}\right)}^{2}\cdot r+1}-1\right]-1$$

(15) $$m=\frac{\frac{f_{c}}{f_{m}}\cdot \left[3\cdot {\phi_{II}}^{2}-{\left(\frac{f_{c}}{f_{m}}\right)}^{2}\right]+2}{{\left(1+\phi_{II}\right)}^{2}}+6\cdot (n-1)\cdot \mu \cdot \frac{{\left(\frac{1}{1+\phi_{II}}-\frac{z_{L}}{h}\right)}^{2}}{\frac{1}{1+\phi_{II}}}$$

(16) $$M=\frac{b\cdot h^{2}}{6}\cdot f_{m}\cdot m$$

were

*f_c_*: compressive strength of wood;

*f_m_*: compressive strength of the reinforcement;

*ϕ_II_*: ratio of the strain on the bottom and top sides for model II;

*µ*: degree of reinforcement;

*z_L_*: distance of the reinforcement element to the edge of the wooden beam;

*n*: ratio of elastic modulus;

*h*, *b*: beam height/width;

*p*, *q*, *r*: auxiliary values;

*m*: factor of increase for the linear-elastic moment capacity, taking into account the amplification and plastic effects in the wood compression area;

*M*: moment capacity of the strengthened beam at the change to the fracture state.

### 2.6. Static System

**Figure 3:**
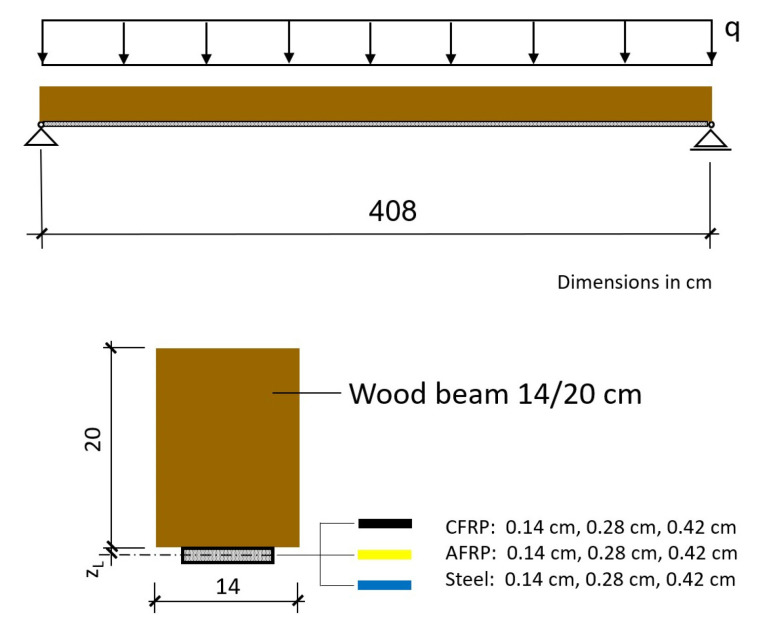
Static system for parameter study.

The calculations were performed in the form of a parameter study with varying reinforcement ratios on a timber beam with practical dimensions and loads. Figure 3 shows the static system, the load arrangement, and the timber and reinforcement cross-section.

The span width was 4 m, and the described beam has an influence width of 1 m, so the results refer to a ceiling width of 1 m. With regard to the determination of the uniform load, a characteristic total load (live load of 3 kN/m^2^ according to ÖNORM B 1991-1-1 [41]) plus the dead load of the floor (0.4 × 0.2 × 4 (wood beam) + 0.04 × 20.5 (filler) + 0.02 × 4 (wood floor) = 1.05 kN/m^2^) without the partial safety factor q = 4.05 kN/m^2^ was assumed. This corresponds approximately to a serviceability limit state (initial deflection) for the conditions shown in Figure 3. Assuming a beam center distance of 1 m, these area-related values then also correspond to the plane uniform load on the wooden beams. The further procedure now deals with the question of the extent to which a significant improvement in the load-bearing behavior can be achieved with an application of generally 10 cm-wide steel, AFRP laminates, or CFRP laminates, which were bonded in single, double, or triple layers. The calculation was designed as a comparative parameter study with practical framework conditions and a variable degree of reinforcement. For reasons of transparency and due to the lack of available partial safety factors for AFRP and CFRP composites used for wood reinforcement, these studies were, on principle, not carried out at the design level, but the elasticity and strength figures were taken as the mean values according to Table 1.

## 3. Results

### 3.1. Internal Forces

**Figure 4:**
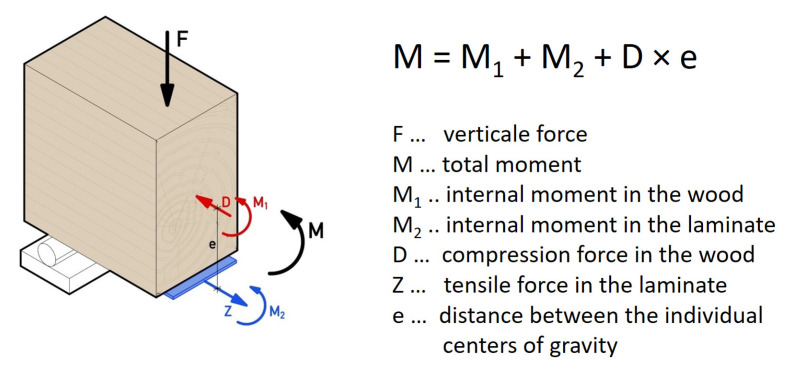
Internal forces on the reinforced timber beam.

In the first step, the internal forces were calculated using the equations given in Section 2.2. Figure 4 shows the distribution of the partial internal forces and their mathematical equation. The composite beam is considered a system with partial internal forces, i.e., a moment and a normal force act in the wood and in the reinforcing laminate. In the present system, the normal force in the laminate appears as a tensile force due to the positive moment load. All these quantities are calculated with the equations of the elastic composite theory according to Natterer and Hoeft [3].

The compressive force D in the wood is the normal force from Equation (2), and the tensile force Z in the lamella has the same value for reasons of balancing.

**Figure 5:**
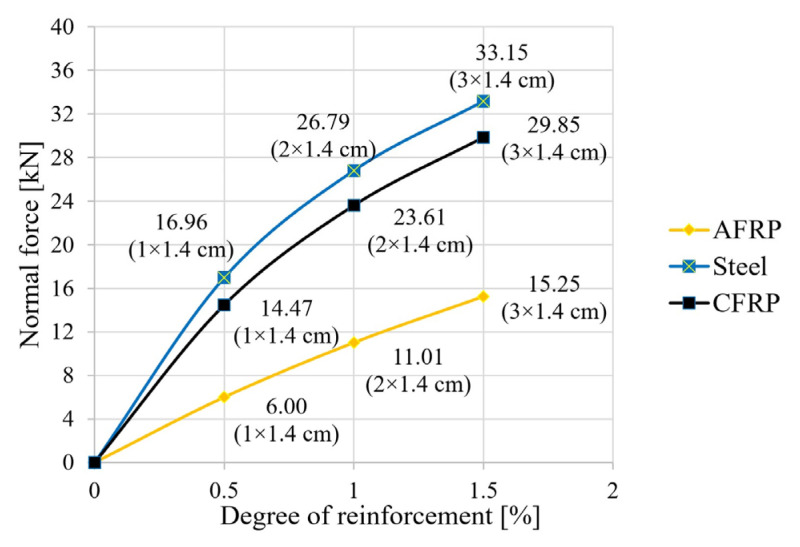
Influence of the reinforcement ratio on the normal force in the reinforcing element.

**Figure 6:**
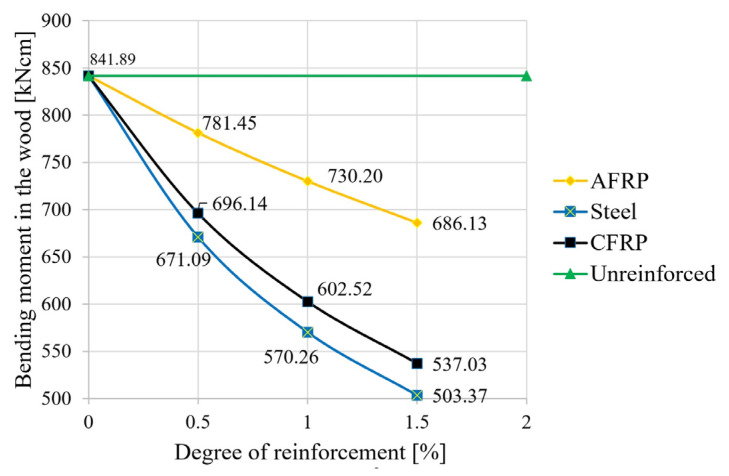
Influence of the degree of reinforcement on the partial moment in the wood.

**Figure 7:**
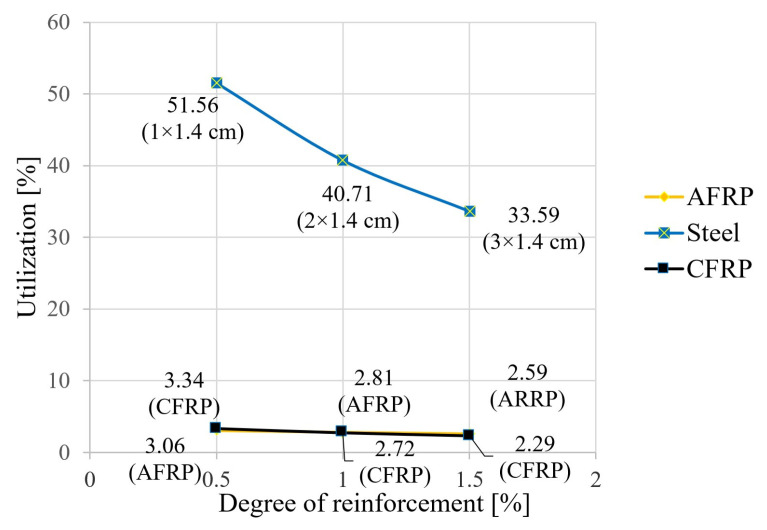
Influence of the degree-of-reinforcement ratio on the tensile member utilization.

The following figures show the results of the calculations for the tensile force in the reinforcing element (Figure 5), the bending moment (Figure 6), and the degree of utilization of the reinforcing element (Figure 7).

The results show that reinforcing timber beams with reinforced steel, AFRP laminates or CFRP laminates led to the establishment of a force couple, with a compressive force in the timber and a tensile force in the laminations of equal magnitude (Figure 5). In the case of CFRP and steel, a tripling of the degree of reinforcement in the size range studied led to a doubling of the tensile force activation. In the case of AFRP, this relationship appeared to be almost linear, with the tensile force activation at a 1.5% reinforcement ratio being only about half that for steel and CFRP (Figure 5).

The pair of forces makes a significant contribution to the total moment and therefore reduces the load on the wooden structure in the form of a decrease in this moment stress (Figure 6). AFRP showed a moment reduction of about 7% compared to that of the raw beam at the minimum reinforcement level and a reduction of about 18.5% at the maximum reinforcement level. This effect was about twice as strong for the steel and CFRP lamellas at the maximum reinforcement level as for the AFRP elements, and it was essentially correlated with the increase in lamella stiffness (see Figure 6; primarily dependent on the geometry and the elastic modulus of the reinforcing member).

With the same geometry, the modulus of elasticity of the reinforcing lamella as the central variable therefore determines the degree of activation of the force couple. A high utilization and economy can only be achieved with the highest possible laminate tensile force and the corresponding strength of the material. This can be demonstrated in particular for steel, with a higher and more favorable degree of utilization compared to CFRP and AFRP here (around 52% at the minimum reinforcement level and around 34% at tripling; see Figure 7). To determine the degree of utilization in the laminate, the effective tensile force in each case is first divided by the laminate cross-sectional area, from which the existing stress in the laminate under the given load follows. The utilization factor is then the ratio of the existing stress in the laminate to the maximum stress (strength) that can be taken up in the laminate. Due to the 13-fold higher strength of CFRP (Table 1), the similarly high tensile force activation of CFRP compared with steel (Figure 7) led to an uneconomical utilization of only 2–3%. The same applies to AFRP laminate, with a similar quotient compared to CFRP at about half the tensile force activation and strength (Figure 5). It is also worth mentioning that the significantly higher strength of CFRP compared to steel (at a similar tensile force) explains the large difference when comparing the differences in the utilization ratios at high and low levels of reinforcement. As shown by the curve run in Figure 7, the utilization ratio dropped significantly throughout the study area, despite increasing tensile force activation. Consequently, the increase in the reinforcement cross-section in the denominator of the corresponding quotient had a greater influence.

### 3.2. Deformations

**Figure 8:**
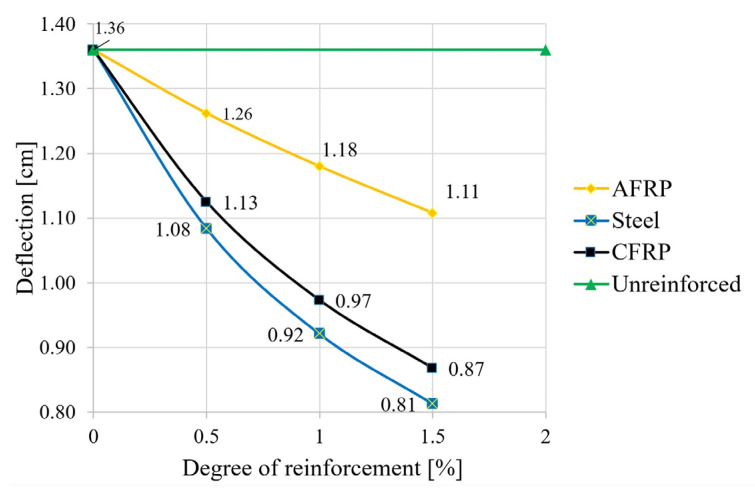
Influence of the reinforcement ratio on the deflection.

**Figure 9:**
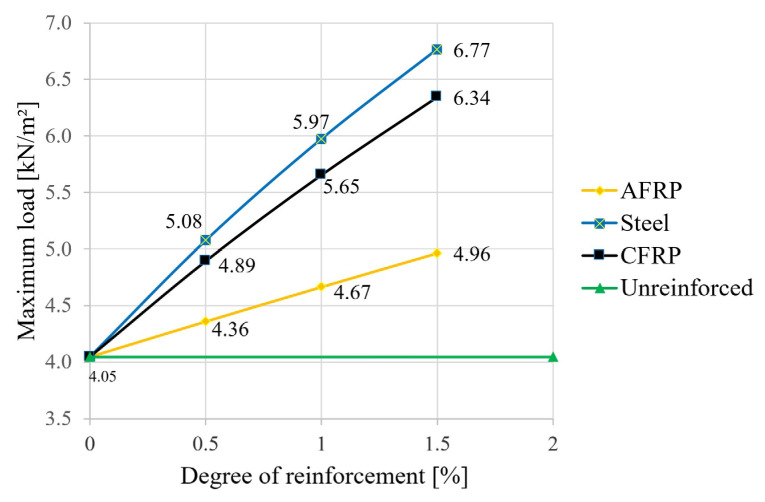
Theoretical load capacity at criterion l/300.

**Figure 10:**
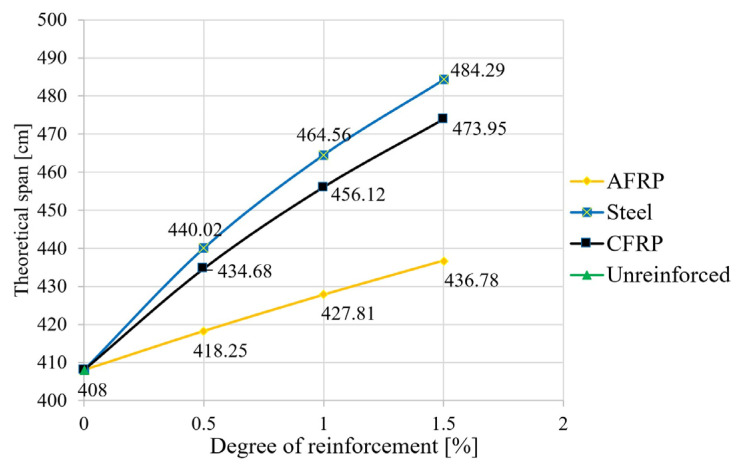
Theoretical span at criterion l/300.

In the second step, the equations given in Section 2 were used to calculate the deformations (deflection in the center of the field). The degree of reinforcement was again varied for the different reinforcement materials. The deflection at a uniform load (Figure 8), the maximum uniform load at l/300 (Figure 9), and the span at a uniform load and at a deflection of l/300 (Figure 10) were calculated.

As shown in Figure 8, a significant reduction in the deflections can also be achieved with reinforcements under the characteristic load combination. In terms of the permitted deformation of the fully utilized reference beam, this means a reduction of 19% for the aramid reinforcement with a triple bond and a reduction of 38% in the deflection for a CFRP or steel application. Thus, in order to achieve full utilization in the case of reinforcement—with the span kept constant—load increases, or, in the case of a constant load, an extension of the span is possible. The results of the corresponding calculations are shown in Figure 9 (increase in load capacity) and Figure 10 (theoretical increase in span).

As can be seen in Figure 9, the reinforcement-induced load reserve was increased by 22% for an aramid reinforcement ratio of 1.5%. This contrasts with the highly significant values of “triple bonding” of an increase of 57% for CFRP and an increase of 67% for mild steel. The dependencies of the load value increase on the reinforcement ratio were almost linear.

Regarding the increase in span length, the increases were lower, with 7% for aramid, 16% for CFRP, and 19% for structural steel (Figure 10). These moderate values can be explained mainly mathematically by the special dependence of the deflection on the span according to the composite equations in Table 2.

### 3.3. Results for the Breaking Condition

**Figure 11:**
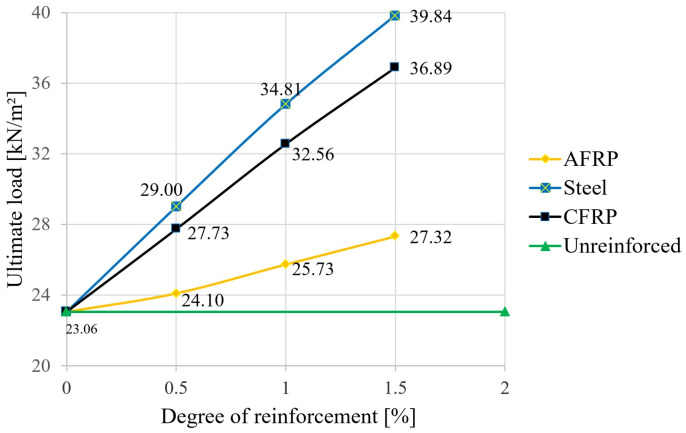
Influence of the reinforcement ratio on the ultimate load—mean values.

In the next step, the failure loads were calculated using the equations given in Section 2. Again, the degree of reinforcement was varied for the different reinforcing materials. Figure 11 shows the results, where the mean values of the material strengths were used.

When determining the ultimate loads, significant increases were also observed for the reinforced variants compared to the unreinforced beam. According to Figure 11, a reinforcement ratio of 1.5% led to an increase in the ultimate load of around 18% for aramid, 60% for CFRP, and around 73% in the case of structural steel. A comparison of Figure 11 with Figure 9 shows that, for the largest degree of reinforcement investigated, the load that would theoretically lead to the maximum allowed deflection can be increased by almost six times for all three materials until failure occurs (increase factor of 5.5 for aramid, 5.8 for CFRP and 5.9 for steel, or 5.7 in the unreinforced case). If one were to refer to the real expected load of 4.05 kN/m^2^ selected in Section 2.2, this could be increased 10-fold to failure at the maximum steel reinforcement level. However, it should be mentioned here that the failure load simulations were based on the average values of the bending tensile strength and compressive strength of the wood. This also explains the significant theoretical ultimate load increase in the unreinforced state (23.06 kN/m^2^) compared to the load that produces the highest deflection (4.05 kN/m^2^ according to Section 2.2).

**Figure 12:**
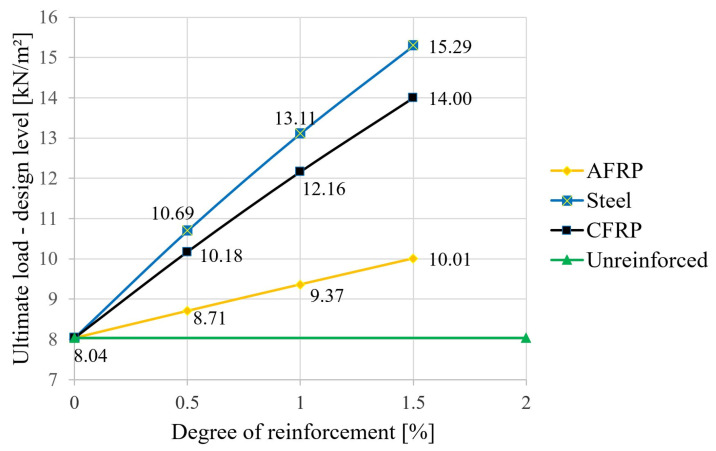
Influence of the reinforcement ratio on the ultimate load—design level.

Based on the semi-probabilistic safety concept, the following results were obtained. Using the characteristic strength values (5% fractile for glulam timber GL24h, f_m,k_ = 24 N/mm^2^ and f_c,0,k_ = 24 N/mm^2^) according to EN 14080 [36], k_mod_ = 0.8 and γ_M_ = 1.25 for glulam timber, and f_m,d_ = f_c,o,d_ = 17.92 N/mm^2^, resulting in the design-level failure load values shown in Figure 12. For the selected loading situation according to Section 2.2 with q_d_ = 3.0 × 1.5 + 1.05 × 1.35 = 7.487 kN/m^2^, this results in a purely mathematical utilization of around 93% for the unreinforced variant. A triple reinforcement with steel significantly increased the load-carrying capacity by almost double. A comparison of Figure 11 and Figure 12 showed relatively clearly that, when safety aspects were included in accordance with an unfavorable case (use of 5% fractile for the wood strengths, including recording the influence of wood moisture and the load application period as well as weighting with partial safety factors), the capacities were only around one-third that of the simulation using the mean values without partial safety factors. However, the increased effect due to the lamellas was verifiable for both concepts (in both cases, slightly less than double, starting from the 3-fold steel bonding).

### 3.4. Environmental Impact

When considering construction techniques, it is essential to take into account not only the mechanical properties, but also the impact on the environment. There are numerous parameters to describe these impacts, such as the global warming potential (GWP), the total non-renewable primary energy demand (PENRT), and the acidification potential of soil and water (AP). All these parameters express important impacts related to the environment. According to [42], the building construction sector is responsible for more than 50% of global greenhouse gases. In this work, since the data on the environmental parameters of aramid are relatively thin, only the global warming potential was considered. This factor indicates the relative contribution of a product to global warming [43]. A consideration of the global warming potential for FRP-strengthening measures for concrete infrastructure can be found in [44]. However, no literature could be found on the wood reinforcements with CFRP, AFRP, and steel described in this paper.

The European standard EN 15804 [45] defines system boundaries that make it possible to assign eco-values to defined processes. It divides the life cycle of a structure into phases A1–D, which describe the manufacturing, construction, use, and disposal phases as well as the reuse potential. At present, the data on aramid bars found in the literature refer only to phases A1–A3, and thus describe the provision of raw materials, the transport, and the manufacturing process. Table 2 shows the data used for the GWP of the materials investigated. It should be noted that the GWP values of the fiber composites refer to rods and not lamellas. However, the influence of this circumstance can be neglected.

**Table 2:** GWP values for the materials used.

Type	Density	GWP	Source
	kg/m^3^	kg CO_2_-eq/kg	
CFRP: Sika CarboDur ^®^ S	1500	12.5	[46]
AFRP: Arapree ^®^	1450	11.2	[46]
Structural steel	7850	1.44	[47]
Concrete	2400	197 ^1^	[48]
Reinforcing steel	7850	0.68	[48]

^1^ in kg CO_2_-eq/m^3^.

**Figure 13:**
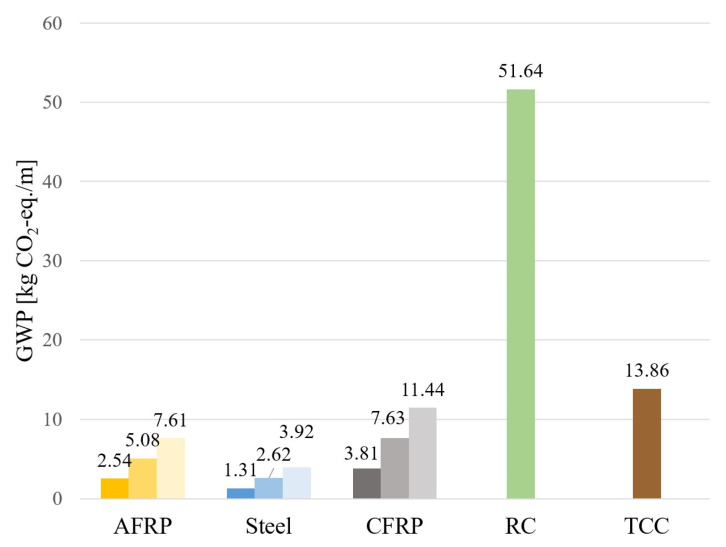
Comparison of GWP values per meter of span including different degree of reinforcement for AFRP, Steel and CFRP.

Since the GWP values are related to the mass of the building materials (with the exception of concrete), the actual geometry must be taken into account for a statement. Figure 13 shows the GWP values of the different building materials, which result when the GWP values per unit mass are multiplied by the geometry of the laminates and the bulk density. In addition to tension member reinforcement measures, a reinforced concrete slab and a TCC slab were also considered in the study. For the reinforced concrete (RC) variant, a slab thickness of 20 cm and a reinforcement content of 90 kg/m^3^ were assumed. The TCC slab was estimated with a top concrete slab (thickness = 6 cm) and a reinforcement content of 50 kg/m^3^.

It can be seen that, with the help of tension member reinforcements, lower GWP values can be achieved than with a concrete layer or a completely new reinforced concrete floor, which comparatively causes about five times as many CO_2_ emissions as the other measures. It should be mentioned that this study is only intended to show a rough overview of CO_2_ emissions. In order to be able to make more precise statements, a more detailed consideration that includes the connecting materials (adhesives and composite screws), the demolition of the existing structure (in the case of the reinforced concrete variant), and the emissions of the entire life cycle is required.

In the following, the results for the lamella reinforcements are considered in detail. For the given geometry of the laminates, the structural steel provided the best result in terms of environmental impact. Lower GWP values can be expected with an aramid lamella than with a CFRP laminate. These results only refer to the geometry of the laminate considered in the parametric study. As already shown in Figure 8, this geometry did not reach the tensile member utilization of aramid and CFRP by far, which is why the cross-section could be chosen to be significantly smaller for these variants. This would result in a lower mass and reduce the GWP values of the variants. In order to take this into account when calculating the environmental impact, Table 3 shows the GWP value determined for a specified geometry; in this case, laminate b/h = 10/0.42 cm, in relation to the tensile strength of the individual materials according to Table 1. In addition, the GWP value is also given in Table 3 in relation to the modulus of elasticity (according to Table 1). Thus, the environmental impact of the individual materials can be concluded, if an increase in stiffness is primarily required.

In view of these correlations, it can be seen that the assessment of the individual building materials in terms of CO_2_ emissions depends on the application area. While structural steel causes the highest GWP values for load-bearing reinforcements, this building material was shown to be the most suitable variant when used as a reinforcement measure for increasing the stiffness values.

Taken as a whole, it can be said that fiber-reinforced composites are more suitable for strengthening the load-bearing capacity in terms of environmental impact, while tensile member reinforcement with the help of structural steel can be considered the most reasonable measure for improving the serviceability property in comparison. Finally, it should be mentioned that the values determined here only refer to the manufacturing process. As a result of the durability of aramid and CFRP, an improvement in the GWP values can be expected over the rest of the life cycle.

## 4. Discussion

The degree of activation is primarily determined by the cross-sectional size and modulus of elasticity of the laminations. Thus, for steel and CFRP laminations with a similar modulus of elasticity, the results of the load-bearing behavior analyses were approximately the same. A high utilization and economic efficiency can therefore only be achieved with the highest possible lamination tensile force and the corresponding strength of the material.

With regard to the permitted deformation of the fully utilized and unreinforced beam, the result was a decrease of 19% for an aramid reinforcement with triple bonding and a decrease of 38% in the deflection for a CFRP or steel application. Thus, the deflection reduction relevant to construction practice can be achieved mostly with triple bonding of steel and CFRP laminations. The analysis of the ultimate loads showed that significant increases can also be achieved with the described reinforcements, both for the actual strength value and at the design level.

In the following, a comparison of the calculation results with other studies, in which experimental tests are described, is carried out. Since the present static system was not applied in the articles, but rather very different test specimens and configurations, the increase of the improvement as a function of the degree of reinforcement can only be determined and compared selectively. Also, the internal forces calculated here cannot be considered, since they cannot be determined in the tests, but only the ultimate loads and the deflections can be considered.

In [49], test specimens 75/300 mm with and without CFRP bars d = 11 mm (not prestressed) applied to the underside were investigated. The degree of reinforcement was 0.42%. In the bending tests (span 570 cm), the stiffness increased from 1.96 to 2.33 × 10^10^ N/mm^2^, which corresponds to 18%. The calculation in this study shows an increase of 20% for the corresponding deflection (1.36 mm to 1.13 mm) according to Figure 8. This is a very good agreement. However, this is not the case for the breaking load, since an unrealistic increase of 64% was measured compared to the non-reinforced beams, but according to Figure 11, only a load increase of 20% was calculated. This is attributed to the small number of specimens and the strongly fluctuating strength values for wood (compression failure).

Also, in [50], FRP and steel-reinforced glulam timber beams are investigated experimentally and theoretically. Also, beams 75/300 mm at a span of 570 cm with different degrees of reinforcement and arrangement of the bars were tested in the bending test. In the evaluation (Figure 27 in [50]), an increase in the bending moment of 28% is given for a degree of reinforcement of 1.0%. According to Figure 11, an increase from 23.06 kN to 32.56 kN, which corresponds to 41%, can be read for the ultimate load, which is equal to the bending moment. Thus, lower improvements were measured in the tests, although the variation of the tests (R^2^ = 0.59) is also relatively large.

A review of several series of experiments published in the literature is given in [16]. The results from up to 14 studies are used and the increases in load capacity in the tests and theoretically are given. Without going into the individual test series here, these range from 6 to 64%. The differences between the measured and theoretical stiffnesses vary depending on the test series and are in some cases very large, as shown in [16], mainly due to the different reinforcement methods and the natural variations of wood strength.

The tests described in [32] showed very good agreement with the measurements from the large-scale bending tests (see Section 2.3). These were 5.2% (average) for the deflections (elastic composite theory according to Natterer and Hoeft) and 5.0% (average) for the breaking loads. The reason for the good agreements can be found in the fact that the modulus of elasticity (as the central input variable for the engineering models) was first determined on each specimen in the unreinforced state.

In summary, it can be said that a comparison with the experiments described in the literature is difficult because of the different configurations of the test specimens, large variation in the measurement results, and, for the most part, a small number of specimens. The best agreement is made with the experiments described in [32], which also corresponds well to the configuration calculated in this article. In summary, it can also be stated that an economically viable utilization of the laminates is possible, predominantly with the steel material. The low utilization rates in the case of CFRP and aramid are due to the significantly higher strength values compared with steel.

## 5. Conclusions

In the case of residential buildings in urban areas, it is necessary to preserve the existing substance to a large extent with regard to ecological and urban developmental aspects. One possibility is to reinforce the underside of wooden beams. Only materials such as reinforcement steel and fiber composites, including carbon-fiber-reinforced polymer (CFRP) laminates and aramid (AFRP) elements, can be considered.

The first main conclusion is that, particularly for steel and carbon-fiber-reinforced polymer (CFRP) reinforcements, significant increases in the ultimate load can be achieved and the often-important deformation behavior can be significantly improved. Especially the steel variant leads to high improvements in deflection and breaking load behavior, with the base material (wood) also being utilized more economically as a result.

With regard to a comparison with test results described in the literature, it is seen that only selective comparisons of the maximum loads and deflections are possible, since the test series have very different configurations, different arrangements of the reinforcements, often small numbers of specimens and widely varying strength values of wood. This can already be seen in the literature review in [16].

So, the second main finding—which is the main topic of this work—is that a comparison of the reinforcements mentioned is only possible if they are examined under the same conditions and all aspects, such as the forces, stresses, deformations, degrees of utilization, etc., are taken into account. We have therefore carried out these calculations for a specific situation described in Section 2.4 under the same conditions. This is to provide engineers and scientists with a basis for the planning and calculation of reinforcements with AFRP, CFRP, or steel, including full details of the required formulas.

In terms of the environmental impact in the form of the global warming potential, it can be said that tension member reinforcement is to be favored in comparison to the installation of a reinforced concrete slab or a TCC slab. The optimal choice of the slab material to be used depends on the type of reinforcement required. While fiber composites are advantageous from an ecological point of view as a reinforcement of the load-bearing properties, lower CO_2_ emissions can be achieved by means of a laminate made of steel if an increase in stiffness and, thus, an improvement in the serviceability properties is required.

## Figures and Tables

**Table 1:** Mechanical properties of the materials—mean values.

Type	Elasticity Modulus	Tensile Strength	Source
	N/mm^2^	N/mm^2^	
CFRP: Sika CarboDur ^®^ S	170,000	3100	Product info [33]
AFRP: Arapree ^®^	60,000	1400	Product info [34]
Structural steel	210,000	235 ^1^	EN 10025 [35]
Glued laminated timber GL 24 h	11,500	51.4	EN 14080 [36]

^1^ yield strength.

**Table 3:** GWP values for the materials in relation to the tensile strength and the modulus of elasticity.

Type	GWP/Tensile Strength	GWP/Elasticity Modulus
	kg CO_2_-eq/m/MPa × 10^6^	kg CO_2_-eq/m/MPa × 10^6^
CFRP: Sika CarboDur ^®^ S	3691	67
AFRP: Arapree ^®^	5438	127
Structural steel	16,695	19

## Data Availability

The original contributions presented in this study are included in the article. Further inquiries can be directed to the corresponding author.
